# Plasma levels of natriuretic peptides and development of chronic kidney disease

**DOI:** 10.1186/s12882-015-0163-9

**Published:** 2015-10-24

**Authors:** Noriyuki Ogawa, Hiroshi Komura, Kenji Kuwasako, Kazuo Kitamura, Johji Kato

**Affiliations:** Frontier Science Research Center, University of Miyazaki, 5200 Kihara, Kiyotake, Miyazaki 889-1692 Japan; Department of Internal Medicine, Circulatory and Body Fluid Regulation, Faculty of Medicine, University of Miyazaki, Kiyotake, Miyazaki 889-1692 Japan; Department of Occupational Therapy, Kyushu University of Health and Welfare School of Health and Science, Nobeoka, Miyazaki 882-8508 Japan

**Keywords:** Natriuretic peptides, Chronic kidney disease, General population, Plasma level

## Abstract

**Background:**

Plasma levels of atrial and brain natriuretic peptides (ANP and BNP) are increased in patients with chronic kidney disease (CKD) complicated with deteriorated kidney function, but the relationship between the plasma level of ANP or BNP and the future development of CKD is unclear.

**Methods:**

We measured the plasma ANP and BNP levels of 294 local residents without CKD in a Japanese community (56.5 ± 10.4 years, mean ± S.D.), who were followed up for the development of CKD over the next 7 years.

**Results:**

Sixty-three residents developed CKD during the follow-up period, and the baseline level of plasma ANP of these residents was significantly higher than in those without CKD development. Kaplan-Meier analysis showed that the residents with higher ANP than the median value developed CKD more frequently than those with lower ANP. The association between plasma ANP level and CKD development was found to be independent of baseline estimated glomerular filtration rate by a Cox proportional hazards model, while this association became insignificant when adjusted by age; plasma ANP was significantly correlated with age. Compared with ANP, the relationship between plasma BNP and CKD development was unclear in these analyses.

**Conclusions:**

Age-related elevation of plasma ANP levels preceded the development of CKD in the general population of Japan, raising a possibility for ANP being involved in the development of CKD.

## Background

The cardiac hormones atrial and brain natriuretic peptides (ANP and BNP) are secreted from the atria or ventricles in response to increased fluid volume or elevated blood pressure [1–6]. These natriuretic peptides act on the kidneys, blood vessels and adrenal glands, where they exert natriuresis, vasodilatation and an inhibitory action on aldosterone secretion, thereby forming a negative feedback loop to reduce fluid volume or blood pressure [3–6]. In recent decades, a number of functional analysis studies have been performed, revealing that ANP and BNP have not only natriuretic or vasodilatory effects but also direct cardiovascular protective actions, such as the inhibition of cardiac fibrosis and the suppression of vascular smooth muscle cell proliferation [3–6]. Their protective roles on the kidneys have also been proposed because ANP and BNP were experimentally shown to inhibit the proliferation of mesangial cells and fibrosis of the kidneys [7–10]. In heart failure patients, augmented secretions of ANP and BNP result in elevated plasma levels of these natriuretic peptides, which have been widely used as markers of disease severity [5, 6, 11]. Similarly, the plasma levels of these two natriuretic peptides or N-terminal fragments of their precursor peptides were reported to be increased in patients with chronic kidney disease (CKD) complicated by impaired renal function, suggesting the possibility that these peptides can be prognostic markers for the progression of CKD or the deterioration of renal function [12–17]. In these reports, substantial numbers of the study subjects were those who had already developed CKD with reduced renal function; meanwhile, there are currently limited data available about the relationship between the future development of CKD and plasma levels of the natriuretic peptides, ANP and BNP, in those without CKD [18, 19]. In the present study, we examined whether the elevation of ANP or BNP levels in plasma is seen prior to the development of CKD by following up local residents without CKD for 7 years.

## Methods

### Study subjects and protocol

Local residents of the Kiyotake area, Miyazaki, Japan, who underwent an annual regular health check-up from 1995 to 1999, were randomly selected for this study. Upon visiting the community center of Kiyotake Town, the medical history of the residents was taken, and blood pressure was measured with an oscillometric automatic device (BP-103iII, Colin, Japan) in a sitting position by experienced nurses. The history taken was confirmed by physicians, who then carried out physical examination including auscultation. Thereafter, urine was collected and blood was drawn from an antecubital vein. Serum lipid and glucose levels were measured using an automatic analyzer (AU2700; OLYMPUS, Tokyo, Japan) with serum creatinine levels determined by an enzymatic method. Residents with a fasting blood glucose level of 126 mg/dL or higher were excluded from this study to avoid analyzing cases of CKD secondary to diabetic nephropathy. In addition, we also excluded those with medical history, symptoms or signs indicative of any heart disease, or with overt cardiovascular diseases, because plasma levels of ANP and BNP are elevated in patients with heart failure.

Residents were judged to have CKD when the estimated glomerular filtration rate (eGFR) was <60 mL/min/1.73 m^2^ or spot urine protein determined by dipstick measurement was ≥ +1 (30 mg/dl). GFRs were calculated using the following formula of the Japanese Society of Nephrology: 194 × serum creatinine^-1.094^ × age^-0.287^ mL/min/1.73 m^2^, further multiplied by 0.739 for women [20]. Of those receiving the check-up during the recruitment period, 294 residents judged to be without CKD (98 males and 196 females; 56.5 ± 10.4 years, mean ± S.D.) were followed up for the development of CKD over the next 7 years at the annual health check-ups.

To measure the plasma levels of ANP and BNP, blood was collected with 1.0 mg/mL EDTA-2Na and 500 kallikrein inhibitory units (KIU)/mL of aprotinin. Plasma was then obtained by centrifugation at 3000 rpm for 10 min at 4 °C and stored at −30 °C until the assay. Plasma levels of ANP and BNP were measured using immunoradiometric assays specific to the two peptides, as previously reported [21, 22].

This study was approved by the Review Committee for Cooperative and Commissioned Research and the Ethics Committee of the University of Miyazaki Faculty of Medicine. All subjects examined gave their informed consent before participating in this study.

### Statistical analysis

All of the data were analyzed with IBM SPSS software version 22.0 (IBM, Armonk, NY, USA). Two groups were compared by either the unpaired *t*-test or the chi-squared test. The rates of CKD development in the local residents with plasma ANP or BNP level higher or lower than the median values were compared by Kaplan-Meier analysis and log-rank test. Both univariate and multivariate Cox proportional hazard models were used to identify factors significantly associated with the development of CKD. In addition, the relationships between plasma levels of the natriuretic peptides and the other parameters were tested by simple regression analysis. All data are expressed as the means ± S.D. and *P* <0.05 was considered to be significant.

## Results

Table 1 shows the basal clinical parameters and plasma levels of ANP and BNP in the residents with or without the development of CKD. During the follow-up period of 7 years, 30 men and 33 women developed CKD, the diagnosis of which was made by eGFR <60 ml/min/1.73 m^2^ in 12 residents, by dipstick proteinuria ≥ +1 in 31, or by both in 20. In the comparison between the residents with and without the development of CKD, the male residents were more susceptible to CKD development than the females. The residents with CKD development were significantly older, showing higher arterial pressure, serum creatinine levels and lower eGFR, than those without CKD development. The plasma level of ANP in the residents who developed CKD was significantly higher than in those who did not, and a similar tendency was observed for the plasma level of BNP, but the difference was not statistically significant.

**Table 1:** Comparison of basal parameters and plasma natriuretic peptide levels between the residents without or with CKD development

	Development of CKD	
	(−)	(+)
Number of subjects (male %)	231 (29)	63 (52)^*^
Age (year)	55.1 ± 10.3	61.9 ± 8.7^*^
Body mass index (kg/m^2^)	22.8 ± 3.1	23.3 ± 3.2
Systolic arterial pressure (mmHg)	124 ± 16	136 ± 21^*^
Diastolic arterial pressure (mmHg)	75 ± 11	80 ± 10^*^
Mean arterial pressure (mmHg)	92 ± 12	99 ± 12^*^
Serum creatinine (mg/dL)	0.58 ± 0.12	0.65 ± 0.13^*^
eGFR (ml/min/1.73 m^2^)	93 ± 16	86 ± 20^*^
Total cholesterol (mg/dL)	197 ± 36	199 ± 29
Fasting blood glucose (mg/dL)	90 ± 8	92 ± 8
ANP (pg/mL)	13.7 ± 7.0	16.8 ± 10.7^*^
BNP (pg/mL)	16.4 ± 14.3	23.0 ± 26.3

*eGFR* estimated glomerular filtration rate, *ANP and BNP* atrial and brain natriuretic peptides

means ± S.D.

^*^ *P* < 0.01, vs. residents without CKD development

**Fig. 1:**
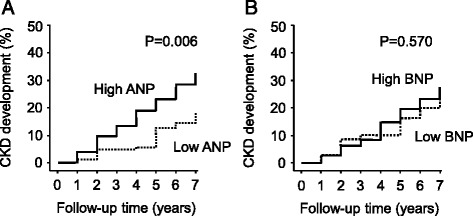
Kaplan-Meier analysis of CKD development in the residents with higher or lower values of ANP (**a**) or BNP (**b**). The subjects were divided into two groups by the median value of ANP or BNP and followed up for 7 years. The differences in the rates of CKD development were evaluated by log-rank test

We then divided the study subjects into two groups of higher and lower ANP or BNP by the medians (ANP, 12.6 pg/mL; BNP 13.1 pg/mL) and compared them using Kaplan-Meier analysis and log-rank test. As shown in Fig. 1, the group with higher ANP showed a significantly higher rate of CKD development than the group with lower ANP. Similarly, the rate of CKD development in the higher-BNP group was slightly higher, but the difference was insignificant compared with those with lower BNP.

The data were further analyzed using univariate and multivariate Cox proportional hazard models, where we used gender, age, mean arterial pressure, eGFR and ANP as explanatory covariates, because these parameters were found to be significantly different between the residents with and without CKD development (Table 1). In univariate analysis, all the parameters listed in Table 2 were judged to be significantly associated with CKD development. Next, we evaluated those parameters by multivariate analyses, the results of which are shown as Models 1 and 2 in Table 3. In Model 1, because eGFR was calculated by using age and serum creatinine, we excluded age from the analysis, identifying all the parameters shown in this model as independently significant factors for CKD development. When we constructed Model 2 including age, both eGFR and ANP became insignificant, and indeed, a significant correlation was noted between plasma ANP and age (*r* = 0.366, *P* < 0.01) by simple regression analysis of the study subjects. Thus age was found to be a powerful cofounder in the relationship between ANP and CKD development. In addition, the ANP level was weakly, but significantly correlated with mean arterial pressure (*r* = 0.148, *P* < 0.05) and eGFR (*r* = −0.130, *P* < 0.05).

**Table 2:** Identification of factors associated with CKD development by univariate analysis

Factors	β	Risk ratio	95 % CI
Gender (M = 1; F = 2)	−0.822	0.439^*^	0.268–0.722
Age (years)	0.067	1.069^*^	1.040–1.100
Mean arterial pressure (mmHg)	0.047	1.048^*^	1.027–1.070
eGFR (ml/min/1.73 m^2^)	−0.029	0.972^*^	0.954–0.990
ANP (pg/mL)	0.030	1.030^*^	1.009–1.052

*eGFR* estimated GFR, *ANP* atrial natriuretic peptide

^*^ *P* < 0.01

**Table 3:** Identification of factors associated with CKD development by multivariate analyses

	Model 1			Model 2		
Factors	β	Risk ratio	95 % CI	β	Risk ratio	95 % CI
Gender (M = 1; F = 2)	−0.698	0.498^**^	0.299–0.827	−0.679	0.507^**^	0.305–0.843
Age (years)				0.042	1.042^*^	1.009–1.077
Mean arterial pressure (mmHg)	0.038	1.039^**^	1.017–1.062	0.031	1.032^**^	1.009–1.055
eGFR (ml/min/1.73 m^2^)	−0.021	0.976^*^	0.961–0.998	−0.013	0.987	0.968–1.006
ANP (pg/mL)	0.026	1.026^*^	1.002–1.052	0.013	1.013	0.986–1.040

*eGFR* estimated GFR, *ANP* atrial natriuretic peptide

^*^ *P* < 0.05

^**^ *P* < 0.01

## Discussion

Both ANP and BNP are cardiac hormones having counter-regulatory roles against increased fluid volume and elevated blood pressure [3–6]. In patients with heart failure, plasma levels of ANP and BNP are progressively elevated in relation to the severity of the disease, so they have been widely used as markers of heart failure [4, 6, 11]. Similarly, plasma levels of the two natriuretic peptides are elevated in patients with chronic renal failure [12, 13], and in recent years, elevated plasma levels of either the natriuretic peptides or the N-terminal fragments of their precursors were shown to be biomarkers for patients with CKD: the higher the plasma levels were, the faster the deterioration of renal function [14–17]. Compared with these reports, there have been limited data available as to the plasma levels of ANP and BNP in the early stage of renal impairment prior to CKD development. Fox et al. revealed that elevation of blood levels of BNP predicted future incident of microalbuminuria by a population-based cohort study [18]. According to Bansal et al., higher levels of an N-terminal peptide of the BNP precursor were associated with rapid decline of renal function and incident CKD [19]. Meanwhile, there have been few reports showing association between plasma levels of ANP and future development of CKD. In the present study, we revealed that age-related elevation of the plasma level of ANP preceded the development of CKD in local residents without overt cardiovascular diseases, while such a phenomenon was unclear for BNP compared with ANP.

First, discussion may be focused on the mechanisms behind the elevation of plasma ANP levels in residents who developed CKD during the follow-up period. In the present study, the baseline levels of plasma ANP were found to be correlated with age, mean arterial pressure and eGFR by simple regression, among which, aging may have a particular importance because the association between the plasma levels of ANP and development of CKD was dependent of age. ANP is metabolized in two different pathways: binding to clearance receptors (NPR-C) and breakdown by the enzyme neutral endopeptidase (NEP) expressed in various organs including the kidneys [5, 6]. It was previously reported that the receptor number of NPR-C on platelets in the elderly decreased, resulting in reduced clearance of ANP from the bloodstream, compared with that in younger subjects [23]. Also, reduction of renal function with reduced activity of peptide hydrolysis may need to be considered because of the correlation between the plasma ANP and eGFR levels at baseline. On the other hand, increased secretion of ANP from the cardiac atria may be a factor: elevation of blood pressure could increase the stiffness of the left ventricle, resulting in elevated left atrial pressure, which augments the secretion of ANP [5, 6]. Thus, it seems to be necessary to take both possibilities into account: a decrease in clearance or breakdown and an increase in secretion, as the mechanism for an elevated plasma ANP level prior to the development of CKD.

Next, we may need to discuss possible roles of ANP, the plasma levels of which were elevated prior to the development of CKD in association with aging in the present study. Both ANP and BNP exert blood pressure-lowering action through natriuresis, vasodilation and the suppression of aldosterone secretion [3–6]. Experimental studies in vitro and in vivo showed that ANP has an inhibitory effect on the proliferation of smooth muscle and mesangial cells, and renal fibrosis, implying a possible kidney-protective role of this bioactive peptide [7–10]. Clinical evidence for therapeutic application of the natriuretic peptides to CKD patients is limited at present, while Sezai et al. showed a possible kidney-protective effect of ANP infused into patients undergoing coronary artery bypass graft surgery [24]. According to a retrospective study by Ng et al., BNP was superior to nitroglycerin in preventing GFR decline in those with heart failure [25]. In the present study, although an elevated BNP level prior to the development of CKD was unclear, we may be able to raise a possibility for ANP in modulating the development of CKD in the general population. Recently, a large clinical trial revealed that the inhibition of natriuretic peptide breakdown by the neutral peptidase (NEP) inhibitor LCZ696 with angiotensin receptor blockade activity improved the prognosis of patients with heart failure [26]. A hypothesis may be raised: further enhancement of the action of ANP could also be a strategy for those at high risk of CKD [27]; however, neither the natriuretic peptides nor NEP inhibitors have been directly proven to be effective in suppressing the development or progression of CKD in humans.

Lastly, limitations of this study should be raised as follows. Plasma levels of ANP or BNP are elevated in patients with cardiac hypertrophy or reduced left ventricular function [3–6]. When recruiting the study subjects, we excluded those with cardiac diseases, such as heart failure and ischemic heart disease, based on the medical history and physical examination by physicians. Meanwhile, it might have been impossible for us to exclude subclinical cardiac conditions affecting the plasma levels of ANP or BNP, because no data were available about echocardiography or chest X-ray. Next, diagnosis of CKD was made based on eGFR values and proteinuria determined by dipstick method, which is not as accurate as quantitative measurement of urinary protein or albumin to creatinine ratio. The third point is the small number of study subjects or those developing CKD, limiting the ability to generalize the present results. Indeed, BNP was not found to be significantly associated with CKD development in this study, but we are unable to exclude possibility of this relationship because of a lack of statistical power. Fourthly, the present data were collected from a Japanese community and evaluated by the eGFR formula for Japanese people. Therefore, it remained to be clarified whether the present results can be applied to other ethnic groups or races.

## Conclusions

Increased levels of plasma ANP, which were associated with aging, preceded the development of CKD in local residents without overt cardiovascular diseases, suggesting a possible role of this bioactive peptide in the development of CKD in the general population of Japan.

## Abbreviations

ANP: Atrial natriuretic peptide
BNP: Brain natriuretic peptide
CKD: Chronic kidney disease
eGFR: Estimated glomerular filtration rate
NEP: Neutral endopeptidase
NPR: Natriuretic peptide receptor
